# Response to Letter to the Editor From Landrigan et al: “Chemicals Used in Plastic Materials: An Estimate of the Attributable Disease Burden and Costs in the United States”

**DOI:** 10.1210/jendso/bvae083

**Published:** 2024-04-24

**Authors:** Leonardo Trasande, Kevin Park, Vladislav Obsekov, Michael Belliveau

**Affiliations:** Department of Pediatrics, NYU Grossman School of Medicine, New York, NY 10016, USA; Department of Population Health, NYU Grossman School of Medicine, New York, NY 10016, USA; NYU Wagner Graduate School of Public Service, New York, NY 10012, USA; Department of Medicine, NYU Grossman School of Medicine, New York, NY 10016, USA; Children's Hospital of Philadelphia, Philadelphia, PA 19104, USA; Defend Our Health, Portland, ME 04101, USA

**Keywords:** plastics, perfluoroalkyl substances, phthalates, bisphenols, disease, cost

We appreciate the comments of Landrigan et al [1] concerning our recent analysis published in the *Journal of the Endocrine Society* [2]. This dialogue is exceedingly important in advancing our understanding of the health and economic consequences of the out of control exposures to chemicals that arise from our plastic society. We also appreciate their acknowledging the Endocrine Society's work [3]. Since 2015, the Endocrine Society has led the way in documenting the disease burden and economic costs due to chemicals used in plastic materials [4]. The estimates would not have been possible without the support of 30 scientists across the globe who meticulously evaluated the best available evidence in humans and model organisms.

Landrigan et al objected to our statement that the Minderoo-Monaco Commission on Plastics and Health assumed all PBDEs, phthalates, and bisphenols are used in plastic manufacture, noting that the Commission stated that “Estimates suggest that over 90% of exposure to these substances comes from plastics.” [5]. We are grateful for this correction. The secondary source they cite for this statement, however, only supports such a conclusion for bisphenols and phthalates, while suggesting without attribution to a primary source that only 80% of exposure to flame retardants and just 1% of exposure to PFAS is attributable to plastics [6].

Our manuscript relied on authoritative sources and a more sophisticated analysis to derive the plastic-related fraction (PRF) of each chemical's use to apply to estimates of disease burden and costs, concluding that 98% of exposure to flame retardants comes from plastics and, for PFAS exposure, 93% of PFOA and 32% of PFOS is plastics related [2]. Clearly, estimates of the plastics-related fraction of chemical use and exposure are important and further work to refine them will continue to improve our recognition of the consequences of plastics.

Landrigan et al report a mathematical error in an estimate of cardiovascular mortality our group had originally published [7]. This error was originally identified by Li and Suh in a comment to *Environmental Pollution* [8]. These authors identified the error easily because our modeling assumptions were transparent, as are underlying calculations to our analysis of the disease burden and costs due to plastics in the United States. In part because we contributed this error in the literature, we felt it was important to highlight the use of this error by the Commission to resolve some differences in overall estimates, and we agree that correction of the Commission report was appropriate.

Considering this, our findings that perfluoroalkyl and polyfluoroalkyl substances, phthalates, bisphenols, and flame retardants contribute substantially to disease, disability, and early death, and $249 billion in associated costs annually in the United States through their use in plastic materials stand as a step up from previous estimates [2]. The Commission has contributed substantially to the plastic pollution discourse in uniting a massive body of evidence to support human health in 1 place [5], and we fully support this open dialogue among scientists to continue to improve our estimates of chemical use and disease burden and the use of these data in global public health decisions.
